# Virgin Coconut Oil in Paste Form as Treatment for Dyspareunia and Vaginal Dryness in Patients With and Without Rheumatic Autoimmune Diseases: An Efficacy and Safety Assessment Pilot Study

**DOI:** 10.7759/cureus.40501

**Published:** 2023-06-16

**Authors:** Marco A Albornoz, Janelle F Burke, Erin K Threlfall

**Affiliations:** 1 Rheumatology, Riddle Hospital, Main Line HealthCare System, Media, USA; 2 Urgent Care, Tower Health, Malvern, USA; 3 Nursing, Thomas Jefferson University, Philadelphia, USA

**Keywords:** rheumatoid arthritis, primary sjogren's syndrome, virgin coconut oil in paste form, vaginal dryness, rheumatic autoimmune diseases, virgin coconut oil, dyspareunia

## Abstract

Introduction

The use of virgin coconut oil (VCO) as an over-the-counter (OTC) treatment for vaginal dryness and dyspareunia (VDD) in the general population has increased worldwide despite the absence of evidence-based studies supporting its efficacy. The principal objective of our pilot study was to scientifically validate the significant benefits and safety of using intra- and peri-vaginal application of VCO in paste form (VCOPF) for the treatment of VDD in patients with and without rheumatic autoimmune diseases (RAD). Additionally, multiple psychosocial, sexuality, and disease activity variables were also assessed.

Methods

A survey study of patients with chronic VDD, with and without RAD, treated with a single proprietary brand of VCOPF via the ‘CocoRelief' protocol continuously for at least six months in an outpatient rheumatology practice setting. We evaluated the comparison of group characteristics, treatment outcomes, and satisfaction questions by Fisher’s exact test or chi-square test for independence for categorical variables and two-sample t-tests for continuous variables.

Results

Of the 53 respondents, 31 (58%) had an RAD and 22 (42%) did not. Rheumatoid arthritis and primary Sjogren’s syndrome comprised 75% of the RAD group. The non-RAD cohort had both a higher baseline mean of intercourse pain (on a scale of 0-5) before VCOPF use (4.4 (SD 1.1) vs 3.9 (SD 1.0) (p = 0.094)) and a higher mean intercourse pain after VCOPF (2.0 (SD 1.3) vs 1.3 (SD 1.1) (p = 0.039)). VDD decreased by 55% in the non-RAD and 66% in the RAD population. Although not statistically significant (p = 0.195), VCOPF was at worst comparable to estrogen-containing therapies (ECT). No adverse events (AE) were documented.

Conclusion

A high percentage of women with VDD, with and without RAD, needlessly continue to experience quality-of-life-altering physical and psychosocial morbidity due to the underutilization and lack of awareness of VCO-containing therapy. The small sample size, non-blinded, non-randomized, pilot platform, and the use of a non-validated assessment tool represent the principal limiting factors. This study revealed that VCOPF, when used in paste form via the CocoRelief protocol, provided statistically significant long-term VDD-related efficacy not inferior to ECT without AE in patients with and without RAD. VCOPF is, therefore, likely to be a useful, cost-effective alternative in a substantial percentage of patients with and without RAD, particularly in women hesitant to utilize ECT due to cost and/or fear of adverse effects.

## Introduction

Vaginal dryness and dyspareunia (VDD) in the pre- and postmenopausal setting are well-recognized features of primary Sjogren’s syndrome and other rheumatic autoimmune diseases (RAD), and it affects approximately 10-20% of the total non-RAD United States population [1-5]. However, the actual prevalence may be significantly higher as 50-60% of women do not inform their medical practitioners of VDD either due to embarrassment and/or lack of awareness, resulting in significant underreporting of the condition [6]. VDD can be associated with severe sexual function impairment, psychosocial distress, and reduced health-related quality of life [6,7]. Existing over-the-counter (OTC), non-estrogen-containing therapies (NECT), including water- and oil-based lubricants and vaginal moisturizers, are often limited by lack of efficacy mainly due to poor lubricity and/or short duration of action. Estrogen-containing therapies (ECT) have demonstrated proven benefits and are considered the gold standard; however, concerns about potential adverse events (AE) and cost are commonly cited as reasons for limiting or eliminating their long-term usage [8-12].

Over a span of seven consecutive years, the principal investigator (PI), a clinical rheumatologist, observed a marked salutary response to the intra/peri-vaginal application of virgin coconut oil in paste form (VCOPF) for the management of VDD in 294 patients with and without RAD. An extensive review of the medical literature failed to identify any well-designed, evidence-based study that evaluated the use of VCOPF as a solitary therapy for VDD in either RAD or non-RAD subjects. However, a small number of peer-reviewed studies, disease-specific websites, and natural health blogs have identified a host of putative virgin coconut oil (VCO)-induced benefits, including, among others, xerostomia and xerosis relief, atopic dermatitis control, hair and cardiovascular protection, prevention of Alzheimer’s, antimicrobial attributes, and anti-inflammatory enhancement [13-23].

The primary objective of this evidence-based study was to evaluate the therapeutic response in pre- and postmenopausal adult women with VDD, both with and without RAD, lasting more than six months, to at least six months of VCOPF usage, and to assess the frequency of treatment-induced AE. The secondary objective was to analyze multiple psychosocial, sexuality, and disease activity variables. Due to the absence of any evidence-based studies examining the use of this novel treatment, a pilot format was utilized. 

## Materials and methods

This study was conducted in Riddle Memorial Hospital, Media, Pennsylvania, United States. Main Line Hospitals Institutional Review Board issued approval for the study (approval number: E-21-5139 dated May 28, 2022).

Study design

This is a 13-question electronic survey study, including a single question that assessed 17 psychosocial, sexuality, and disease activity variables, of 67 patients with pre-existing VDD, with and without a RAD, previously treated with VCOPF for at least six months, and observed during a consecutive six-month period in an outpatient solo private practice rheumatology setting. A random numeric patient code was exclusively assigned by and available only to the PI. VCO, when utilized in the treatment of VDD, is commonly applied in liquid form. In our patients, VCO was applied only in paste form. This transformation occurs when VCO, which is typically housed in a jar or travel packet box, is spared from direct sun exposure and stored at a temperature less than 72°F. For purposes of uniformity, and based on the PI’s prior positive clinical experience, the Trader Joe’s® Organic Virgin Coconut Oil brand (Trader Joe's, Bellingham, Washington, United States) was solely utilized in all study patients. The PI’s pre-existing patients taking VCOPF were included in the study subject to the ongoing exclusive use of the study-selected brand of VCO. The PI and co-investigators have no past or present relationship with the maker of the VCO utilized in the study nor have they derived any direct or indirect compensation from them. Topical placement of a small amount of VCOPF to the volar forearm was required prior to the first vaginal application to screen for AE. The standard dose of VCOPF was two to four teaspoons manually applied intra- and peri-vaginally immediately prior to coitus. We have labeled this intervention as the ‘CocoRelief' protocol. Due to anecdotal reports of possible VCO-induced damage to the structure of a latex-containing condom, VCOPF usage in this setting would disqualify the patient from study participation. Additional exclusion criteria included the use of a non-study brand of VCO, oral or topical allergy to coconut, and pregnancy. An anonymized, low-risk treatment consent form/instructional handout was provided to and signed by all study participants. A gynecologic examination was never performed by the PI, either as it relates to this study or in the past or present execution of his duties as a rheumatologist. 

Statistical analysis

A total of 67 patients were surveyed regarding their experience with VCOPF as a treatment for VDD. Of those, 53 respondents completed at least 80% of the questions and were included in the analysis. The participants were grouped by RAD status (yes or no). Symptom stratification was determined using a non-validated six-point VDD activity scale created by the PI. Seventy-six percent of the RAD population had rheumatoid arthritis and primary Sjogren’s syndrome. We then compared group characteristics, treatment outcomes, and satisfaction questions by Fisher’s exact test or chi-square test for independence for categorical variables, and two-sample t-tests for continuous variables. As this is a pilot study, we chose a p-value of <0.1 as significant. All analyses were performed with Stata 17.0 (Statacorp, LLC, College Station, Texas, United States).

## Results

A total of 22 (41.5%) participants did not have an RAD, whereas 31 (58.5%) did. The majority of participants had persistent vaginal dryness (96.2%) and persistent vaginal pain (92.4%). Most patients were 50 years or older (83%) and post-menopausal (83%). There was a difference in age between the two groups, with the non-RAD group being younger than the RAD group (p = 0.098). A lower percentage of the RAD patients (74%) were postmenopausal compared to the non-RAD group (95%, p = 0.064) (Table 1). 

**Table 1:** Patient Characteristics and Pain Scores *Statistically significant. RAD:  Rheumatic autoimmune disease.

	RAD			
	No	Yes	Total	
	n = 22	n = 31	n = 53	p
Persistent vaginal dryness				1
No	1 (4.6%)	1 (3.2%)	2 (3.8%)	
Yes	21 (95.4%)	30 (96.8%)	51 (96.2%)	
Persistent vaginal pain				0.633
No	1 (4.6%)	3 (9.7%)	4 (7.6%)	
Yes	21 (95.4%)	28 (90.3%)	49 (92.4%)	
Age group				0.098*
18-29	0 (0)	1 (3.2%)	1 (1.9%)	
30-39	1 (4.6%)	3 (9.7%)	4 (7.6%)	
40-49	1 (4.6%)	3 (9.7%)	4 (7.6%)	
50-59	6 (27.3%)	16 (51.6%)	22 (41.5%)	
60-69	13 (59.1%)	7 (25.6%)	20 (37.7%)	
70-79	1 (4.6%)	1 (3.2%)	2 (3.8%)	
Menopausal status				0.064*
Premenopausal	1 (4.6%)	8 (25.8%)	9 (17.0%)	
Postmenopausal	21 (95.4%)	23 (74.2%)	44 (83.0%)	
Mean intercourse pain before coconut oil use	4.4 (1.1)	3.9 (1.0)	4.1 (1.1)	0.094*
Mean intercourse pain after coconut oil use	2.0 (1.3)	1.3 (1.1)	1.5 (1.2)	0.039*
Mean difference in pain (after - before)	-2.4 (-3.0)	-2.6 (-3.1)	-2.5 (-2.9)	0.568
Adverse events				NA
No	22 (100%)	31 (100%)	53 (100%)	
Yes	0	0	0	

**Figure 1:**
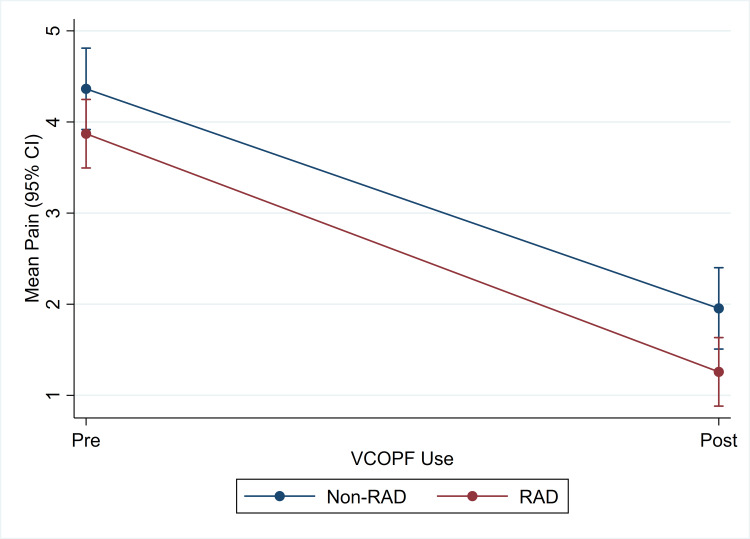
Intercourse Pain by VCOPF Use and RAD Status VCOPF: virgin coconut oil in paste form;  RAD: rheumatic autoimmune disease.

The non-RAD group had a higher baseline mean of intercourse pain (on a scale of 0-5) before VCOPF use (4.4 (SD 1.1) vs 3.9 (SD 1.0), p = 0.094) and had a higher mean intercourse pain after VCOPF (2.0 (SD 1.3) vs 1.3 (SD 1.1), p = 0.039). VCOPF reduced intercourse pain in 55% of the non-RAD and 66% of the RAD populations, both of which were statistically significant. After calculating the mean differences in pain before and after VCOPF use, there was no difference between the groups. No participant experienced AE from the use of VCOPF (Figure 1). 

When comparing VCOPF with other non-coconut oil or non-estrogen vaginally applied products, 94% of the RAD group reported that VCOPF was significantly or moderately superior compared with 77% of those in the non-RAD group (p = 0.195). There were no significant differences for those who previously used ECT. As concerns the 17 psychosocial, sexuality, and autoimmune disease activity variables, 100% responded positively. Statistical significance was found only in the 84% of RAD patients who reported an improved relationship with a spouse or partner compared to the 68% of non-RAD participants (p = 0.097). In 94% of the RAD participants, near-statistical significance was seen relative to an improved duration of vaginal moisture compared to 76% of participants without an RAD (p = 0.104). In total, 83% and 87% of participants stated that VCOPF improved vaginal dryness and duration of vaginal moisture, respectively. In addition, 23% and 19% of participants with RAD reported that VCOPF improved dryness of mouth and eyes compared to 9.1% and 9.1% of non-RAD participants. Twenty-six percent of participants with RAD reported that VCOPF improved work-related productivity compared with 9% of the non-RAD group. Lastly, 45% of participants with RAD reported that VCOPF improved their autoimmune disease activity (Table 2).

**Table 2:** VCOPF Use Satisfaction and Comparison to Other Products by RAD Status VCOPF: Virgin coconut oil in paste form; RAD: rheumatic autoimmune diseases.

	RAD			
	No	Yes	Total	
	n = 22	n = 31	n = 53	p
Q9 Comparison to non-coconut oil or non-estrogen vaginally applied products n (%)				0.195
VCOPF is superior	17 (81.0%)	29 (96.7%)	46 (90.2%)	
VCOPF is comparable	3 (14.3%)	1 (3.3%)	4 (7.8%)	
VCOPF is inferior	1 (4.8%)	0 (0)	1 (2.0%)	
N/A	1	1	2	
Q10 Comparison to intravaginal estrogen applied therapy n (%)				0.579
VCOPF is superior	15 (79.0%)	15 (88.2%)	30 (83.3%)	
VCOPF is comparable	3 (15.8%)	2 (11.8%)	5 (13.9%)	
VCOPF is inferior	1 (5.3%)	0 (0)	1 (2.8%)	
N/A	3	14	17	
Q14 Did VCOPF during intercourse improve or have a positive effect on any of the following? (Yes Only) n (%)				
Vaginal dryness	17 (77.3%)	26 (86.7%)	43 (82.7%)	0.468
Duration of vaginal moisture	16 (76.2%)	29 (93.6%)	45 (86.5%)	0.104±
Libido	14 (63.6%)	19 (61.3%)	33 (62.3%)	1
Ability to obtain an orgasm	15 (68.2%)	19 (61.3%)	34 (64.2%)	0.467
Intensity of orgasm during intercourse	12 (54.6%)	16 (51.6%)	28 (52.8%)	0.247
Texture and appearance of skin	5 (22.7%)	10 (33.3%)	15 (28.9%)	0.201
Dryness of mouth	2 (9.1%)	7 (22.6%)	9 (17.0%)	0.284
Dryness of eyes	2 (9.1%)	6 (19.4%)	8 (15.1%)	0.563
Relationship with spouse/partner	15 (68.2%)	26 (83.9%)	41 (77.4%)	0.097*
Prevented breakup with spouse/partner	2 (9.1%)	4 (13.3%)	6 (11.5%)	0.359
Depression	7 (33.3%)	11 (35.5%)	18 (34.6%)	0.546
Anxiety	9 (40.9%)	7 (22.6%)	16 (30.2%)	0.319
Irritability	5 (22.7%)	9 (29.0%)	14 (26.4%)	0.775
Work-related productivity	2 (9.1%)	8 (25.8%)	10 (18.9%)	0.135
Quality of work-related duties	3 (13.6%)	5 (16.7%)	8 (15.4%)	0.696
General quality of life	16 (72.7%)	23 (74.2%)	39 (73.6%)	1
RAD activity	0 (0)	14 (45.2%)	n/a	< .0001>

Of the 17 psychosocial, sexuality, and disease activity variables in patients with and without RAD who responded positively to the use of VCOPF, nine were greater than expected, three were expected, one was less than expected, and four were uncertain expectations(Table 3).

**Table 3:** Principal Investigator’s Levels of Expectation to Positive Responses to VCOPF Involving 17 Psychosocial, Sexuality, and Disease Activity Variables in Patients With and Without an RAD ¥ RAD patients; ∞ non-RAD patients; *statistically significant. VCOPF: Virgin coconut oil in paste form; RAD: rheumatic autoimmune disease.

Sexuality, psychosocial, and disease activity variables	Greater than expected		Expected		Less than expected		Uncertain	
	Vaginal dryness		Depression		Anxiety		Irritability	
	¥ 87%	∞77%	¥ 36%	∞33%	¥ 23%	∞ 41%	¥ 29%	∞23%
	Duration of vaginal moisture *		Ability to obtain an orgasm		Prevented breakup with spouse/partner		Work-related productivity	
	¥ 94%	∞76%	¥ 61%	∞68%	¥ 13%	∞ 9%	¥ 26%	∞9%
	Libido						Quality of work-related duties	
	¥ 61%	∞64%					¥ 17%	∞14%
	Intensity of orgasm during intercourse							
	¥ 52%	∞55%						
	Rheumatic autoimmune disease activity							
	¥ 45%	n/a						
	General quality of life							
	¥ 74%	∞73%						
	Relationship with spouse/partner *							
	¥ 84%	∞68%						
	Xerostomia							
	¥ 23%	∞9%						
	Xeropthalmia							
	¥ 19%	∞9%						
	Texture & appearance of skin							
	¥ 33%	∞23%						

## Discussion

This pilot study, in addition to reaffirming that VDD is frequently associated with meaningful physical and psychosocial morbidity, validates the efficacy and safety of VCOPF in the management of VDD in patients with and without RAD. The use of VCOPF decreased intercourse-mediated pain in a statistically significant manner by 55% in non-RAD patients and 66% in those with an RAD. However, the differences between the RAD and non-RAD groups as concerns pain before and after VCOPF use did not achieve statistical significance. Other statistically significant results included the following: (a) a higher percentage (83%) of all respondents were above 50 years of age and postmenopausal; (b) a difference in age between the two groups, with the non-RAD group being younger than the RAD group; (c) a lower percentage of the RAD patients (74%) were postmenopausal compared with the non-RAD group; and (d) a markedly improved relationship between spouse or partner favoring the RAD population. Importantly, no minor or major VCOPF-induced AE were documented in any study participant. Although not statistically significant, the use of VCOPF in the combined RAD and non-RAD populations versus those treated with NECT and ECT demonstrated a 59% significant and 28% moderate reduction of VDD in the former, whereas in the latter the reduction in VDD was 23% significant and 34% moderate. Only 2% of the combined non-RAD and RAD patients found VCOPF to be moderately inferior, and 0% found it to be significantly inferior to NECT. None of the patients felt that VCOPF was moderately inferior, and 1% of patients described it as being significantly inferior to ECT. These noteworthy data suggest that the use of VCOPF is, at worst, comparable to ECT and, at best, effective in assuaging the financial burden and its perceived health risks. 

An analysis of a subset of 17 secondary variables among RAD and non-RAD patients revealed that VCOPF either improved or had a positive effect on multiple psychosocial, sexuality, and disease activity components in 100% of patients. Due to the absence of a validated comparator group or study, the findings were categorized, based on the PI’s personal experience with the therapeutic use of VCOPF in VDD, as being: (a) greater than expected; (b) expected; (c) less than expected, and (d) uncertain (Table 3). Of these, the 45% improvement in autoimmune disease activity was the most surprising but not supported by the historical, physical examination, laboratory, and diagnostic imaging findings encountered by the PI. When managing patients with most types of RADs, fatigue, although subjective, is considered a key variable that often accurately mirrors disease activity. The impressive VCOPF-mediated benefits found in several variables in the RAD cohort, particularly those dealing with sexuality and romantic relationships, engendered an enhanced sense of well-being and quality of life. The net effect was that fatigue reduction was very likely a consequence of non-RAD, VCOPF-mediated factors rather than a true reduction of disease activity. An improvement in the duration of vaginal moisture reached near-statistical significance. Despite the lack of statistical significance, when RAD and non-RAD patients were compared, the former demonstrated improvement in the texture and appearance of the skin (RAD 33% vs non-RAD 23%), xerostomia (RAD 23% vs non-RAD 9%), and xeropthalmia (RAD 19% vs non-RAD 9%). During the six-month study period, both groups demonstrated high levels of sustained positive results relative to vaginal dryness (RAD 87% vs non-RAD 77%), general improvement in quality of life (RAD 74% vs non-RAD 73%), and the ability to obtain orgasm (RAD 61% vs non-RAD 67%) but without reaching statistical significance. 

 Although VCOPF in the management of VDD was proven to be moderate to significantly effective and safe in the RAD and non-RAD populations and demonstrated improvement in multiple secondary variables, the small sample size, non-blinded, non-randomized, pilot platform, and use of a non-validated assessment tool limit our ability to draw authoritative conclusions. Yet, as the first study to examine this worldwide problem in a scientifically rigorous fashion, we conclude that VCOPF as a treatment for VDD in patients with and without RAD, when administered via the CocoRelief protocol, will effectively fill an existing therapeutic void and prove to be a highly attractive long-term therapeutic option due to its safety, affordability, and an efficacy comparable to ECT. Moreover, distinct psychosocial and sexuality variables responded positively to VCOPF, resulting in improvement in multiple life-impacting factors. Due to the anticipated increase in female life expectancy in industrial nations, the prevalence of VDD is likely to rise thereby augmenting the demand for effective VDD treatments [24]. A follow-up study determining the duration of therapeutic efficacy from the initiation to the discontinuation of VCOPF in a large cohort is currently underway. Larger multi-center, methodologically sound studies are required to confirm our findings.

## Conclusions

This study demonstrates that VCOPF, when used in paste form via the CocoRelief protocol, provided statistically significant long-term VDD-related efficacy not inferior to ECT, without AE, in patients with and without RAD. It also revealed that a high percentage of women with VDD, irrespective of RAD status, needlessly experience life-altering physical and emotional distress, and interpersonal conflicts due to a lack of effective therapeutic options. Our study has exposed a clear unmet need for new therapies and the modification of existing treatments in the management of patients with VDD with and without RAD.
